# Collaborative Practices in Mental Health Care: A Concept Analysis

**DOI:** 10.3390/healthcare13151891

**Published:** 2025-08-02

**Authors:** Eslia Pinheiro, Carlos Laranjeira, Camila Harmuch, José Mateus Bezerra Graça, Amira Mohammed Ali, Feten Fekih-Romdhane, Murat Yıldırım, Ana Kalliny Severo, Elisângela Franco

**Affiliations:** 1 Graduate Program in Collective Health, Federal University of Rio Grande do Norte, Natal 59078-900, Brazil; jose.graca.121@ufrn.edu.br (J.M.B.G.); elisangela.franco@ufrn.br (E.F.); 2 School of Health Sciences, Polytechnic University of Leiria, Campus 2, Morro do Lena, Alto do Vieiro, Apartado 4137, 2411-901 Leiria, Portugal; 3 Centre for Innovative Care and Health Technology (ciTechCare), Polytechnic University of Leiria, Campus 5, Rua das Olhalvas, 2414-016 Leiria, Portugal; 4 Comprehensive Health Research Centre (CHRC), University of Évora, 7000-801 Évora, Portugal; 5 Department of Postgraduate Nursing, State University of Maringá, Avenida Colombo, 5790-Campus Universitário, Maringá 87020-900, Brazil; camilaharmuch@gmail.com; 6 Department of Psychiatric Nursing and Mental Health, Faculty of Nursing, Alexandria University, Smouha, Alexandria 21527, Egypt; amiramali@alexu.edu.eg; 7 Faculty of Medicine of Tunis, Tunis El Manar University, Tunis 1007, Tunisia; feten.fekih@gmail.com; 8 The Tunisian Center of Early Intervention in Psychosis, Department of Psychiatry, Ibn Omrane, Razi Hospital, Manouba 2010, Tunisia; 9 Department of Psychology, Faculty of Science and Letters, Ağrı İbrahim Çeçen University, Ağrı 04100, Türkiye; muratyildirim@agri.edu.tr; 10 Psychology Research Center, Khazar University, Baku AZ1000, Azerbaijan; 11 Department of Collective Health, Federal University of Rio Grande do Norte, Natal 59078-970, Brazil; kalliny.severo@ufrn.br

**Keywords:** collaboration, mental health, mental health service, interprofessional relations, person centered care, concept analysis

## Abstract

**Background/Objectives:** Collaboration in mental health care is essential for implementing a model oriented towards the psychosocial rehabilitation of people based on multifaceted interventions involving different actors and sectors of society to respond to demands. Despite the benefits presented by the scientific evidence, there are still many barriers to collaborative care, and professionals continue to struggle in reorienting their conduct. The current situation demands organization and the framing of well-founded action plans to overcome challenges, which in turn requires a detailed understanding of collaborative practices in mental health care and their conceptual boundaries. A concept analysis was undertaken to propose a working definition of collaborative practices in mental health care (CPMHC). **Methods:** This paper used the Walker and Avant concept analysis method. This includes identifying the defining concept attributes, antecedents, consequences, and empirical referents. A literature search was carried out from November 2024 to February 2025 in three databases (Medline, CINAHL, and LILACS), considering studies published between 2010 and 2024. **Results:** The final sample of literature investigated consisted of 30 studies. The key attributes were effective communication, building bonds, co-responsibility for care, hierarchical flexibility, articulation between services, providers and community, monitoring and evaluating of care processes, and attention to the plurality of sociocultural contexts. **Conclusions:** This comprehensive analysis contributes to guiding future research and policy development of collaborative practices in mental health, considering the individual, relational, institutional, and social levels. Further research is possible to deepen the understanding of the production of collaborative practices in mental health in the face of the complexity of social relations and structural inequities.

## 1. Introduction

Mental health has gained increasing relevance in the analysis of the health status of populations, whether due to the recognition of its importance for well-being and social development or due to the dissipation of stigma and social exclusion that for years contributed to the marginalization of patients. Today, the role that mental health plays in the functioning of societies and in physical health itself is evident. In a 2024 study, approximately half of the 31 countries surveyed considered mental health to be one of the main health problems faced by the population [1]. According to the World Health Organization (WHO), in 2019, almost one billion people lived with some diagnosable mental disorder, which corresponded to approximately 13% of the global population. In the first year of the COVID-19 pandemic (2020), there was an estimated increase of 28% and 26% in depressive and anxiety disorders, respectively [2]. Other problems, such as stress, panic, somatization disorder, sleep disorders, emotional disorders, post-traumatic stress disorder, and suicidal behavior, also increased significantly [3,4]. The impact of these disorders is multiple and includes a reduction in people’s quality of life, an increase in years of life lost due to health problems, disability, and premature death, in addition to social and economic losses [1,5,6,7,8]. In this sense, care practices focused on people with mental disorders are essential, for which it is necessary to promote intervention in community contexts and ensure the guarantee of rights [9]. In the last century, several countries witnessed a paradigm shift: from a biomedical, hospital-centric model focused on the disease to achieve adaptation of individuals through medicalization and other alienating methods [10], to a model that proposes a broader understanding of health processes—directly influenced by individual, family, and sociocultural factors—and oriented towards the psychosocial rehabilitation of people through multimodal interventions and with the involvement of different actors and sectors of society to respond to demands [11,12,13,14,15,16].

Collaboration in mental health care is a fundamental aspect in achieving this transition between care models. A set of approaches related to Collaborative Practices in Mental Health Care (CPMHC) have been developed in different contexts, considering different components and using different concepts of interdisciplinary and collaborative practice [17]. Some of them are aimed at the integration between professionals specialized in mental health and primary care professionals; others emphasize the relationships between different healthcare disciplines; others value collaboration between healthcare and other sectors, such as education, culture, and social assistance; others address person-centered decision-making; and there are still those that engage and work with communities [18,19,20,21,22,23].

Historically, CPMHP has been focused on healthcare professionals collaborating to deliver comprehensive care across various settings. More recently, this concept includes community members, families, and caregivers, focusing on addressing health needs within the broader community context, emphasizing person-centeredness and shared decision-making [24]. Evidence shows the benefits of CPMHP, with emphasis on improvements in access to mental health services, in the quality of care provided, in clinical outcomes (e.g., adherence to the therapeutic regimen), and in the autonomy and satisfaction of beneficiaries [25]. Recently, the WHO launched the “Guidance on mental health policy and strategic action plans”, which reinforces intersectoral collaboration and the construction of inclusive systems as a way to achieve effective interventions in the field of mental health [11]. Despite the guidance from official bodies and the legislation of some countries, in addition to the successful experiences documented in scientific literature, many barriers to the implementation of collaborative practices are still observed, such as inadequate funding and lack of organizational support. In practice, professionals continue to have difficulty reorienting their conduct [14,26]. Presently, there is a need for organization and construction of action plans, as well as a detailed understanding of CPMHC and its conceptual boundaries. Although the WHO presented a general definition of collaborative practices for the health field in 2010, this definition does not address the specificities inherent to mental health, resulting in a polysemy perceived in published studies [27]. In the field of mental health, collaborative practice involves the interaction between professionals and service users and their families, and management processes within and between systems [26]. However, the interchangeable use of multiple terms, such as “interprofessional work”, “teamwork”, “collaborative work”, “collaborative care”, and “interprofessional collaboration”, can hinder the dynamics of cooperation in mental health services [28,29].

Considering the importance of social, economic, and cultural factors related to mental illness and their impact on the lives of individuals and communities [15], increasingly effective collaborative strategies are needed that broadly address mental health issues, seeking to guarantee basic rights and promote health and social justice—a perspective that is aligned with the Sustainable Development Goals for 2030, in terms of health and well-being and reduction of inequalities [30]. Deepening knowledge about the concept of CPMHC can provide clues to overcome barriers and direct the actions of managers, professionals, users, family members, and communities. Thus, the main objective of this study is to develop a conceptual analysis of Collaborative Practices in Mental Health. In this regard, our main research question is, “What antecedents, attributes, and consequences define the Collaborative Practices in Mental Health Care concept?” Comprehensive knowledge of collaborative practices in mental health care is essential for informing future research and evidence-based practices.

## 2. Materials and Methods

### 2.1. Study Design

This concept analysis follows eight interactive steps described by Walker and Avant [31] (see Table 1). Each stage is crafted to facilitate the transformation of an abstract phenomena into a substantive definition, characterized by features sufficiently practical to inform actions that enhance healthcare quality and patient safety.

### 2.2. Study Details

In order to identify the aforementioned characteristics related to the concept, an integrative literature review was carried out. The search for articles was carried out using three databases (Medline, CINAHL, and LILACS) and occurred on 10 November 2024, with an update on 20 May 2025. To this end, a combination of keywords using Boolean operators and truncation (*) was used in the following search strategy: (collaborat*[Title/Abstract] OR “interprofessional relations”[MeSH Terms] OR “interprofessional relations”[Title/Abstract] OR interdisciplinary[Title/Abstract] OR multidisciplinary[Title/Abstract] OR “patient-centered”[Title/Abstract] OR integrative[Title/Abstract] OR intersectoral[Title/Abstract] OR “decision making, shared”[MeSH Terms]) OR “decision making, shared”[Title/Abstract] AND (“mental health”[Title/Abstract] OR “mental health service*”[Title/Abstract] OR “community mental health centers”[MeSH Terms] OR “community mental health centers”[Title/Abstract] OR “community psychiatry”[MeSH Terms] OR “community psychiatry”[Title/Abstract]). The search in the LILACS database used the corresponding terms from the Health Sciences Descriptors (DeCS).

### 2.3. Eligibility Criteria

Original articles, primary or secondary, published between 2010 and 2024 were included—considering the publication, in 2010, of the “Framework for Action on Interprofessional Education & Collaborative Practice” by the WHO as an important milestone for the application of concepts in subsequent studies [27]. In addition, we included studies involved people aged 18 or over; that addressed two or more disciplines/areas of knowledge; that were written in Portuguese, Spanish or English; and that were available in full text. Opinion articles, letters to the editor, dissertations, and theses and those that did not respond to the purpose of the concept analysis were excluded.

### 2.4. Data Analysis

Data from the articles included in the final sample of the concept analysis were extracted using an excel spreadsheet containing information about the sociodemographic characterization of the studies and the antecedents, attributes, and consequences of collaborative practices in mental health identified in each study. This instrument was developed and validated by the group of researchers responsible for carrying out the analysis. Four researchers (E.P.; C.H.; J.M.G.; C.L.) carried out the blind screening and selection of studies, as well as the evaluation and data extraction. Inconsistencies were discussed within the research team to reach a consensus.

A thematic analysis was performed to analyze data from the selected articles, following the steps of reading, coding, and theming [32]. Themes emerged from the data via an inductive coding process, validated by the research team composed of mental health professionals and faculty members with expertise in conducting research in the field of mental health. It was therefore possible to organize the information present in the studies in order to identify the antecedents, attributes, consequences, and empirical referents of the concept. After achieving data saturation, extracted data were categorized and labeled.

## 3. Results

**Figure 1:**
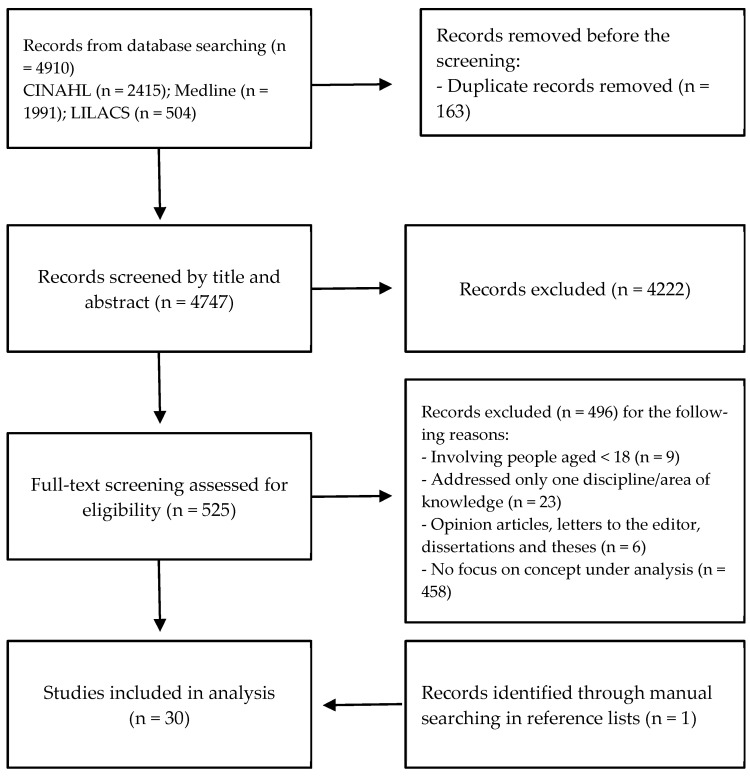
Flow diagram illustrates selection of sources.

A total of 4910 publications were identified through the database search, of which 163 were excluded for being duplicates (see Figure 1); 4222 articles were excluded after screening titles and abstracts. Thus, 525 articles were carefully read to determine their eligibility. From these, 496 articles were eliminated, leaving 29 articles that were included in the final sample. One further article was obtained by examining the reference lists of the papers. The final sample included 30 articles (Supplementary Table S1).

The included studies were carried out in the USA (n = 11), United Kingdom (n = 5), Canada (n = 3), Norway (n = 3), Brazil (n = 2), South Africa (n = 1), Israel (n = 1), Belgium (n = 1), Japan (n = 1), and Australia (n = 1). Regarding the type of study, there were 10 qualitative studies, 3 quantitative studies, 2 mixed methods studies, 4 systematic reviews, 2 scoping reviews, 7 narrative reviews, and 1 concept analysis. Regarding the description of the users or types of treatment investigated, the following were addressed: general/unspecified mental illness (n = 18), severe mental illness (n = 6), depression and/or anxiety (n = 3), special population (such as the older people, students, substance use and women with perinatal depression) (n = 1), and eating disorders (n = 1).

### 3.1. Definitions and Uses of the Concept

The Cambridge dictionary defines “collaboration” as “the act of working together with other people or organizations to create or achieve something” [33]. According to Critical Care Nursing Clinics of North America “collaborative practice” is defined as “an interprofessional process for communication and decision-making that enables the separate and shared knowledge and skills of care providers to influence synergistically the client care provided” [34]. In 2010, the WHO proposed a definition of collaborative practices for the health field in general as those that occur when “multiple health professionals from different professional backgrounds work together with patients, families, caregivers and communities to provide the highest quality of care” [27] (p. 7). According to the Canadian Interprofessional Health Collaborative, “Collaborative practice occurs when healthcare providers work with people from within their own profession, with people outside of their profession and with patients/clients and their families” [35]. In the field of mental health, collaborative practices represent a shift from specialist-centered models to partnerships in which professionals work with patients, not on them, supporting self-care and recovery goals that are meaningful to the person [36].

The Collaborative Care model (CoCM) is the most often referenced framework for integrated mental health care, with more than 80 randomized clinical trials supporting its efficacy across multiple psychiatric conditions [37]. CoCM has demonstrated the capacity to enhance access to behavioral health services, provide patient-centered behavioral and physical health care within a unified environment, and improve overall clinical results [25]. Five essential components are crucial for realizing these benefits: population-based care, measurement-based care, care management, psychiatric consultation, and short evidence-based psychotherapy [37].

### 3.2. Attributes

According to Walker and Avant [31], attributes are the characteristics or aspects that constitute a concept and that allow its broad perception. Seven main attributes of CPMHC emerged from the literature and were organized into three domains (individual/relational, organizational, and contextual) (see Table 2).

In the individual/relational domain, the following attributes emerged: (a) effective communication, which reflects the importance of dialogue and the exchange of information to enable the occurrence of collaborative practices, including empathetic and transparent communication and the exchange of information among all those involved in the care process (users, family members, professionals, etc.) [38,39,40,41,42,43,44,45,46,47,48,49,50,51,52,53,54,55,56,57,58,59,60,61]; (b) building bonds that develop from coexistence and established relationships of trust and that consolidate the sense of belonging, which is applicable for interprofessional teams as well as users and family members [41,43,44,45,51,52,53,54,59,60,62]; (c) co-responsibility for care, which refers to the sharing of responsibilities among professionals with different expertise, and with users and family members, and generally starts with the establishment of common goals and is essential for the development of effective therapeutic plans [38,41,45,46,47,50,52,53,54,55,56,60,62,63].

In the organizational domain, the following attributes emerged: (a) hierarchical flexibility, which refers to power relations and the efforts needed to make them balanced, so that all subjects involved can be heard and there is openness to adapt to situations [40,41,43,47,49,50,52,53,56,60,62,63,64,65]; (b) articulation between services, providers, and the community: CPMHC involves the integration of professionals from different health services (primary care services, specialized care services, emergency and urgent care services) and/or services from other sectors (social assistance, culture, education), in addition to the broader community, wherein the possibility of integrating actors and services according to the needs of users is important [38,39,40,41,42,43,47,49,50,51,52,53,54,55,57,58,59,60,61,62,63,64,65,66]; (c) monitoring and evaluating care processes is essential to achieving collaborative practices. Such evaluations should occur on a regular basis and in a structured manner through the development of continuous improvement projects [38,40,43,47,48,49,57,58,60];

In the contextual domain, a single attribute emerged: attention to the plurality of sociocultural contexts where care takes place. Through culturally congruent and competent care, it is possible to reduce inequities and thereby safeguard the rights of vulnerable people from different groups/populations [38,41,47,57].

### 3.3. Antecedents

Antecedents are the preceding events or incidents, that is, those that must occur or be present before the occurrence of the concept [31]. In the present concept analysis, the identified antecedents were synthesized and organized into the individual/relational, organizational and contextual spheres. In the individual/relational sphere, individual characteristics and skills (role awareness, confidence, expertise) stand out, as well as the relational skills of professionals in contact with people with mental illness, family members, and other professionals [39,48,52,64]. At the organizational level, the concept is preceded by the existence of legislation and support policies [41,43,46,52]; adequate funding of mental health structures [38,41,43,49,55,60,61]; interprofessional and transprofessional education [41]; existence of decision-making flows and definition of responsibilities [41,43,44,49,53]; continuing education plans for professionals [38,49,52,53,54,55,64]; and the existence of a formal collaborative culture [39,41,43,45,47,48,49,50,54,60,63,65]. Regarding the contextual sphere, knowledge of the territory of action and the real needs of users and family members emerges [39,41,52,53,57,62].

### 3.4. Consequences

The consequences, in turn, are the results—what occurs as a result of the concept [31]. With the implementation of CPMHC, consequences for people with mental illness include improvement in clinical outcomes and/or recovery of well-being [38,39,41,42,49,52,55,60], prevention of crises and the number of hospital admissions [39], reduction in treatment time [38], promotion of autonomy [41,53,56,60], and user satisfaction [39,53,55]. Consequences for professionals include the valorization of teams [53,56], improvement in the quality of care [39,52], professional satisfaction [39,52], retention of human capital [39], and strengthening of professional identity [52,55]. In organizational terms, the change from traditional disease-focused care models to person-centered care is effective [52,53,65] and associated with cost management efficiency [38,42,52,55,60]. Finally, in contextual terms, the connection with the community is strengthened [41,53,60]; stigma and discrimination are reduced [55,56], and consequently there is greater social inclusion [56].

### 3.5. Empirical Referents

Empirical references are classifications of phenomena that, through their existence, illustrate the manifestation of the concept itself [31]. Thus, producing evidence regarding CPMHC is essential to understand the points that need to be improved in each context of action, as well as to identify potentialities. Without standardized instruments that allow the measurement of the concept, it becomes difficult to assess. Thus, the revised Collaborative Practice Assessment Tool (CPAT) was identified, which aims to evaluate collaborative practices in mental health and is composed of five axes: “patient/community-centered care”, “collaborative communication”, “interprofessional conflict”, “clarification of roles”, and “environment” [67]. This scale is composed of 21 items and evaluates the effectiveness of interprofessional teams in mental health and suggests optimal solutions for enhancing team performance. Additionally, it contributes to the generation of more robust evidence for collaborative practices in mental health settings globally. Additional research is necessary to further assess the reliability and validity of the CPAT as a comprehensive scale.

### 3.6. Case Examples

Following the approach of Walker and Avant [31], a model, borderline and contrary case was developed. The cases were adapted from real-world examples taken from professional experiences of mental health service workers. Ethical approval was unnecessary because all names attributed to the characters are fictitious, and no personal information was used.

#### 3.6.1. Case Model

A model case illustrates all the key features of the specified concept [31]. Michael, a 35-year-old black man, was referred to a community mental health service for alcohol use disorder. There was an initial meeting with Michael and the team members. During the meeting, Michael mentioned that he had been treated at another service for bipolar disorder and had stopped taking his medication. The psychologist contacted the health unit and spoke with the psychiatrist who had treated Michael. In his report, the psychiatrist shared that since his mother’s death (nearly a year ago), Michael had not worked and had no family support. As a result of this conversation, the team worked with social services to facilitate the receipt of financial support and support for mourning. During the time he spent at the community mental health service, Michael built important bonds with the team, having developed greater autonomy and responsibility for his own care and coming to understand the reasons that caused the crises leading to his alcohol consumption. The psychologist worked in direct coordination with the psychiatrist, pharmacist, and nurses of the service in the management of the therapeutic regimen. Michael actively participated in both the negotiation and the definition of goals. Michael attended the service’s therapeutic groups with other users, where they developed various socio-emotional regulation activities (e.g., visual arts, theater, therapeutic writing, etc.). Michael also attended other health services, through referrals, when he needed care in other areas of health. Over time, he began to show less impulsivity, greater understanding of his health status, and reduction of alcohol consumption and the risk behaviors related to it. The team defined a follow-up and monitoring plan, with home visits to assess adherence to the therapeutic regimen and the process of social reintegration.

#### 3.6.2. Borderline Case

Borderline cases include most, but not all, of the distinguishing attributes of the concept studied [31]. Stella, a 23-year-old woman, was diagnosed with Personality Disorder and Panic Disorder. Without adequate treatment, she frequently sought emergency care due to impulsive behavior and attempts at self-harm. Stella had previously been seen by a psychiatrist, but she was not adhering to her medication regimen due to side effects of the therapy. The emergency room team contacted a specialized outpatient mental health service in her area for a referral. She was received and scheduled a psychiatric and psychological consultation. Sometime after the follow-up at the service, Stella took the medications as prescribed and had less frequent crises. This was possible thanks to the establishment of a relationship of trust between her and the psychiatrist. The professional listened to Stella’s reservations and fears regarding the side effects of the medications, and together, they discussed the best pharmacological strategies. Stella also had a good relationship with the psychologist, with whom she met once a week, and with the professionals responsible for the therapeutic workshops she attended. The team discussed the case with Stella and identified the need to mediate a connection between her and her mother, who had been estranged for some time because she did not know how to deal with her daughter’s emotions. Stella agreed to invite her mother to some meetings, and little by little, the bond between them was reestablished. At a certain point, the team concluded that Stella could start being monitored by the Primary Health Care team. Stella and her mother did not agree with this decision, claiming that, despite the improvements observed, she still had difficulty leaving the house, participating in social activities and even working. Stella said that she would feel lost and abandoned if she was unable to attend the service. The Primary Care team had difficulty monitoring Stella, since the professionals claimed that they did not have sufficient knowledge about mental disorders. Over time, Stella was unable to establish connections with her community (work, leisure, etc.), so she began to have intense crises again and was taken to the emergency room. There was no safe transition of care between the outpatient clinic and the primary health care team. Furthermore, the primary care professionals, due to their lack of knowledge about mental illness and its consequences, did not provide users with adequate support.

#### 3.6.3. Contrary Case

Eric, 24, unemployed, was admitted to a psychiatric unit after a severe psychotic episode associated with the use of cannabinoids. Despite the complexity of the condition, care was provided in a fragmented manner, with no coordination between professionals. Initially, Eric was treated in the emergency room, where mechanical and chemical restraint was administered to control his aggression and agitation. After controlling his symptoms, he was referred to psychiatric hospitalization. During his hospitalization, Eric recovered his ability to gain insight and requested support from psychology and social services. However, monitoring was only conducted by the medical team, which did not coordinate with the other team members to define an integrated care plan—only a few specific meetings with Eric to comply with data collection protocols. There were no discussions with the patient and family to define medium- and long-term stabilization strategies or to facilitate the user’s social reintegration after discharge. Furthermore, Eric and his family did not receive adequate guidance on the disorder and on the forms of support needed after discharge. After some time, Eric was discharged with a complex medication regimen, but no contact was established with the community mental health team for follow-up. As a result, a week later, he returned to the emergency department with a new psychotic episode and suicide attempt, due to the lack of an integrated care plan. All defining attributes are missing in this contrary case [31].

### 3.7. Operative Definition

**Figure 2:**
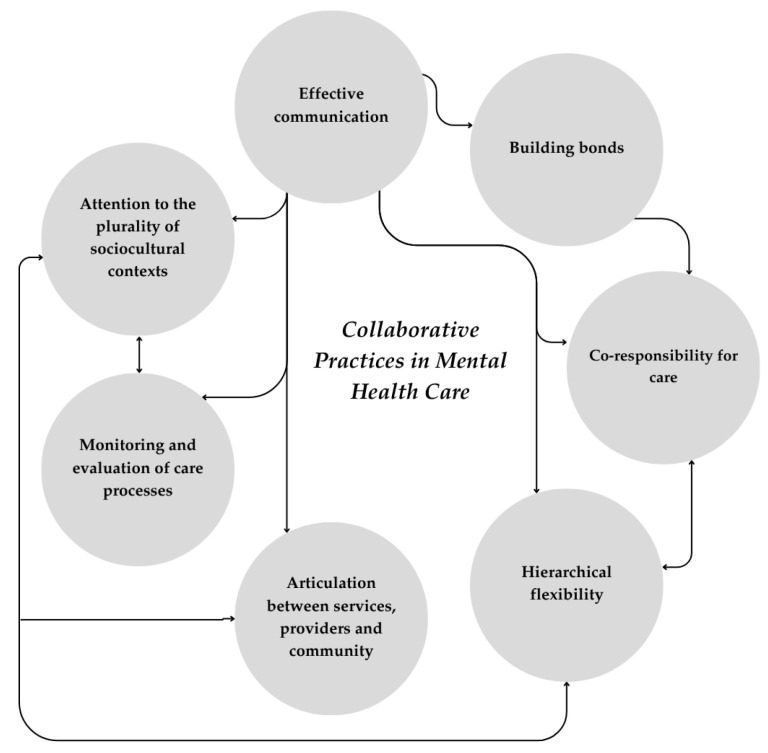
Conceptual representation of collaborative practices in mental health care (CPMHC).

After the attributes of the concept were identified, a new definition emerged (see Figure 2). Thus, the concept of collaborative practices in mental health corresponds to a dynamic interpersonal process involving interdisciplinary knowledge and practices that, in partnership, share responsibilities in planning, implementing, and evaluating mental health care that meets the multidimensional needs of users and thereby promotes quality of care and health gains. This process is facilitated through effective and transparent communication, with the ongoing exchange of information and shared decision-making in which the user and their caregivers are the center of the care process. Such integration implies organizational structures guided by hierarchical flexibility, democratic decision-making flows, and ethical and cultural sensitivity capable of responding to the complexity of the challenges posed by mental illness. Health professionals must be cognizant of and embrace their roles, expertise, and obligations within participating disciplines. Effective collaborative practices are attained by interprofessional education, updated guidelines, regular meetings, and a referral mechanism.

## 4. Discussion

This concept analysis identified seven core attributes for the CPMHC concept: (a) effective communication, (b) building bonds, (c) co-responsibility for care, (d) hierarchical flexibility, (e) articulation between services, providers and the community, (f) monitoring and evaluating the care processes, and (g) attention to the plurality of sociocultural contexts. These attributes reveal a broader understanding of mental health, which considers the subjectivity and relationships between people involved in care processes and foresees cooperation between different sectors of society (health, social assistance, education, culture, justice, among others). While revealing essential aspects of CPMHC, these attributes signal the importance of offering care based on the local reality of services and communities [38]. Producing and using evidence on the procedures carried out in the daily routine of services is important to take advantage of opportunities for improvement, overcome obstacles, and obtain better clinical, institutional, and social results. However, the experiences of people with mental health needs should not be neglected [68,69]. Adherence to any guidelines or models needs to be critically analyzed, and interventions must be aligned with the needs of patients and families. Therefore, the production of participatory assessments stands out as a way to build indicators that reflect what is actually relevant to those who are simultaneously targets and co-producers of care [70,71,72].

The antecedents of the concept demonstrate that CPMHC are influenced by a range of factors, such as the characteristics and skills of professionals (role awareness, confidence, expertise, relational skills); the existence of legislation and support policies; adequate funding of mental health structures; interprofessional and transprofessional education; the existence of decision-making and responsibility definition flows; continuing education plans for professionals; the existence of a formal culture of collaboration; knowledge of the territory of action and the real needs of users and families. Encounters between people seems to favor exchanges and collaboration. Some studies point to the importance of co-location (the practice of integrating mental health services into non-specialized settings, such as primary health centers or schools, rather than maintaining these services isolated in traditional mental health settings), but being in the same care environment does not, in itself, guarantee favorable outcomes [73,74,75]. Information and Communication Technologies (ICTs) can be used to amplify communication and the exchange of information. Efficient and integrated information systems optimize working time, and telephone contacts can be useful for quick consultations between professionals and quick contact with users for information or check-ups, for example [76]. Above all, it is essential that there be an organizational (and interorganizational) structure with agreements, in-person and remote flows, and/or protocols that allow and encourage these movements [45,47,60].

The positive consequences of CPMHC include improved clinical outcomes and/or promotion of mental well-being; prevention of crises and the reduced number of hospital admissions [38,39,41,42,49,52,55,60]; reduction in treatment time [38]; promotion of autonomy [41,53,56,60]; user satisfaction [39,53,55]; valorization of teams [53,56]; improvement in the quality of care [39,52]; professional satisfaction [39,52]; retention of human capital [39]; reinforcement of professional identity [52,55]; transformation of traditional disease-centered care models into person-centered care [52,53,65]; efficiency in cost-effectiveness with little to no net increase in healthcare costs [38,42,52,55,60]; strengthening of the connection with the community [41,53,60]; reduction of stigma and discrimination [55,56]; and greater social inclusion [56]. The negative consequences of failing to implement these collaborative practices include the lack of partnership between users/family members [54], and conflicts related to professional performance and identities caused by the lack of definition of roles and responsibilities [50].

Within the scope of CPMHC, apparently less emphasis has been given to the influence of social markers that impact the relationship of subjects with society, that is, intersectionality [77,78]. Some studies mention power relations between users and professionals and the need to pay attention to the plurality of sociocultural contexts [38,41,47,57], but gender identity, race/ethnicity, sexual orientation, and social class are also factors that deserve consideration when seeking to understand inequities as barriers to CPMHC. Consequently, an individual’s difficulties in maneuvering through the healthcare system as a transgender person, a person of color, and a person experiencing mental distress, cannot be simplified to a singular axis of discrimination; they must be addressed comprehensively, taking into account the intricacies introduced by interrelated power structures [79]. Pervasive exposure to adverse social determinants undermines an individual’s ability to maintain good mental health, recuperate from mental health issues, and avert future mental illnesses [15,80]. In this sense, intersectionality seems to be an important concept to be explored in this field. By recognizing the complexity of mental health experiences, intersectionality allows the development of policies and practices that are more sensitive to the needs of different social groups [79,81].

### 4.1. Study Limitations

The study of concepts and thematic categorization exemplifies the predominant attributes identified in contemporary academic literature, which serves as a strength in addressing current challenges to optimal patient outcomes. The targeted search methodology across three databases, coupled with the restriction to literature published within the last 14 years, may present constraints. Even though intentionally broad, it is possible that the search string was not sensitive enough to retrieve all articles related to the concept under analysis. In addition, the language restriction to Portuguese, English, and Spanish may also have left some studies inaccessible. It is also important to investigate the meanings and interpretations attributed to the concept of CPMHC in different cultural and professional contexts. The capacity of CPMHC to address the distinct issues of particular demographics (including students, displaced people, ethnoracial minoritized groups, older patients, women’s health, and substance abuse treatment) warrants further examination, as historically, diagnosing and treating mental health disorders in these groups has proven challenging. Moreover, further research is necessary to validate the relationships among the antecedents, attributes, and consequences of CPMHC and to clarify the methods for structuring collaborative practices among people and organizations. This approach establishes a foundation for creating instruments to objectively assess the impact of CPMHC. It also prompts practitioners to recognize and comprehend the facilitators and obstacles to the implementation of successful collaborative practices in mental health settings. A mixed-methods longitudinal research design incorporating an interprofessional education intervention will most effectively assess antecedent development and evaluate subsequent progress in CPMHC.

### 4.2. Implications for Practice

The personal characteristics and relational skills of professionals play an important role in the implementation of CPMHC [39,48,52,64]. Knowledge about the territory of action and the needs of users and family members is essential for this type of work to be carried out satisfactorily [39,41,52,53,57,62]. Therefore, strategies that define the profile of users and mental health professionals are encouraged, as well as implementing activities to recognize the territory to provide culturally competent care. Safe environments and spaces that promote proximity between users and health teams, conflict mediation and the development of therapeutic relationships should also be implemented. At the same time, organizations must establish communication channels that, on a regular basis, allow them to anticipate difficulties and promote an effective partnership of care [39,41,43,45,47,48,49,50,54,60,63,65]. Investing in continuing education actions focused on the principles of mental health intervention and collaborative practices are necessary [38,41,49,52,53,54,55,64]. Additionally, organizations must create opportunities for employees to foster their well-being to create professionals who are more resilient in the face of mental health challenges. Academies must also ensure that curricula allow for pedagogical practices that encourage simulation and immersive practices in a real context, in order to stimulate the acquisition of collaborative and teamwork skills. Finally, it is important to ensure that legislation and public policies adopt an intersectional approach and are aligned with the perspectives of person-centered care, deinstitutionalization and the strengthening of community-based services oriented towards psychosocial rehabilitation [41,43,46,52].

## 5. Conclusions

In the current scenario of fragmented care, CPMHCs are necessary to provide comprehensive care. This concept analysis indicated that CPMHCs should be guided less and less by self-interest or bureaucratic norms and should base their intervention on interaction and interdependence. This should be achieved through assertive forms of communication, coordination of mental health services, and development of networking strategies, according to the different contexts. The definition of attributes, antecedents, and consequences of CPMHCs in this study will be useful in drawing more precise conceptual boundaries and, thus, contribute to mitigating the gaps between theory and practice, in addition to fostering the development of CPMHC policies, considering individual, relational, organizational, and contextual factors. More research is needed to deepen the understanding of the production of CPMHCs in the face of the complexity of social relations and structural inequities.

## Figures and Tables

**Table 1:** Eight-step analysis model proposed by Walker and Avant [31].

Steps	Description
1. Select a concept	To choose a concept based on its relevance to the research and to further theoretical developments in the field of study
2. Determine the aims or purposes of analysis	To establish why the concept analysis will be carried out and what are its possible implications
3. Identify all uses of the concept that you can discover	To include all uses of the concept from as many different sources as possible
4. Determine the defining attributes	To reveal the cluster of attributes that are most frequently associated with the concept and that allow the broadest insight into the concept
5. Identify a model case	To present an example of the use of the concept that includes all critical attributes of the concept
6. Identify borderline and contrary cases	To present additional cases, such as those that contain most of the defining attributes of the concept being examined but not all of them (borderline cases) and those that meet none of the critical attributes (contrary cases)
7. Identify antecedents and consequences	To identify antecedents (those events or incidents that must occur or be in place prior to the occurrence of the concept) and consequences (outcomes of the concept).
8. Define empirical referents	To identify means by which it is possible to recognize or measure the defining characteristics or attributes

**Table 2:** Concept-defining attributes of CPMHC, organized in the relational, organizational, and contextual domains.

Domains	Attributes
**Individual/Relational**	Effective communication [38,39,40,41,42,43,44,45,46,47,48,49,50,51,52,53,54,55,56,57,58,59,60,61];
	Building bonds [41,43,44,45,51,52,53,54,59,60,62];
	Co-responsibility for care [38,41,45,46,47,50,52,53,54,55,56,60,62,63];
**Organizational**	Hierarchical flexibility [40,41,43,47,49,50,52,53,56,60,62,63,64,65];
	Articulation between services, providers and community [38,39,40,41,42,43,47,49,50,51,52,53,54,55,57,58,59,60,61,62,63,64,65,66];
	Monitoring and evaluation of care processes [38,40,43,47,48,49,57,58,60];
**Contextual**	Attention to the plurality of sociocultural contexts [38,41,47,57]

## Data Availability

All data generated or analyzed during this study are included in this article.

## Supplementary Materials

The following supporting information can be downloaded at: https://www.mdpi.com/article/10.3390/healthcare13151891/s1, Table S1. Characteristics of the studies included in the review (n = 30).
