# Supplementary material for: Collaborative Practices in Mental Health Care: A Concept Analysis
Source: Healthcare (Basel). 2025 Aug 2;13(15):1891. doi: 10.3390/healthcare13151891 (PMC12346314; doi:10.3390/healthcare13151891)
Supplement: Supplementary file 1 [file healthcare-13-01891-s001.zip › healthcare-3763479-supplementary.pdf]

Table S1. Characteristics of the studies included in the review (n=30)

#	Author(s)/ Year / Country	Article type/ Methodology	Sample	Aims	Key findings
[38]	Reist et al. (2022) USA	Narrative review	N/A	This review discusses current research on the effectiveness and cost-efficiency of Collaborative Care. The paper discusses its ability to adapt to specific patient populations, such as geriatrics, students, substance use, and women with perinatal depression, as well as the significance of measurement-based care and mental health screening in achieving improved clinical outcomes.	Current data suggests that Collaborative Care may significantly improve patient outcomes and time-to-treatment in all reviewed settings, and successfully adapts to special patient populations. Despite the high upfront implementation burden of launching a Collaborative Care model program, these costs are generally offset by long term healthcare savings.
[39]	Tomizawaa et al. (2017) Japan	Scoping review	Methodological characteristics extracted from 12 articles.	To present a new conceptual framework for the assessment of interprofessional teamwork.	The findings revealed that structural issues comprised three elements: professional characteristics, client-care characteristics, and contextual characteristics in organisations. Process issues comprised two elements: team mechanisms and community-oriented services. Finally, outcome issues comprised the following elements: clients' outcomes and professionals' outcomes.
[40]	Reilly et al. (2024) United Kingdom	Systematic review	Eight randomised controlled trials (1165 participants).	To assess the effectiveness of collaborative care approaches in comparison with standard care (or other non-collaborative care interventions) for people with diagnoses of severe mental illness who are living in the community.	This review does not provide evidence to indicate that collaborative care is more effective than standard care in the medium term (at 12 months) regarding quality of life, mental state and psychiatric admissions. No differences were shown in quality of life, mental state or admissions to a psychiatric hospital at 12 months. One study showed an improvement in disability at 12 months. Disability was used as an indirect measure of how well people function in their lives, in terms of their social roles and activities. Most of the studies included did not meet a strict definition of collaborative care (what we called type A collaborative care) and there were large variations in the interventions delivered. Furthermore, the majority of evidence was either low- or very low-

					certainty.
[41]	van Rensburg & Brooke-Sumner (2023) South Africa	Scoping review	36 papers were included for analysis.	To identify and describe multi and intersectoral approaches underpinning community-based severe mental illness (SMI) recovery interventions in low- and middle-income countries.	Examples of multi- and intersectoral action included collaboration between healthcare and community support systems, collaboration in providing supported housing and supportive community spaces for recovery, and linkages between biomedical and social spheres of care. Barriers included the dominance of mental health professions in delivering care, and community-based stigmatising attitudes towards SMI. Multi- and intersectoral collaboration for SMI recovery requires investments in financing, education and coordination by a governing body.
[42]	Hernandez et al. (2024) USA	Systematic review	16 articles were included in the sample as they met the inclusion criteria.	To understand the impacts of the Collaborative Care Model on symptom management, diverse populations, and sustainability in healthcare systems	An analysis revealed that, in 12 of the final articles, the CCM led to a statistically significant improvement in anxiety and depression symptoms with viable implementation and sustainability strategies. The CCM is an effective method for improving patient symptoms and can be potentially affordable in healthcare systems.
[43]	Thomson & Chatterjee (2024) United Kingdom	Rapid evidence review	34 reviews and 21 grey literature reports fitted inclusion criteria of adult physical/mental health outcomes/multiple morbidities.	To determine barriers and enablers of integrated care across the United Kingdom	Thematic analysis revealed six themes (collaborative approach; costs; evidence and evaluation; integration of care; professional roles; service user factors) with 20 subthemes including key barriers (cost effectiveness; effectiveness of integrated care; evaluation methods; focus of evidence; future research; impact of integration) and enablers (accessing care; collaboration and partnership; concept of integration; inter-professional relationships; person-centered ethos).

[44]	Gatesy-Davis et al. (2022) USA	Qualitative	39 formal and informal leaders in vocational rehabilitation (n=16), child mental health (n=13), and adult mental health (n=10) systems.	To describe collaborative activities that support better vocational services for transition-age youth with serious mental health conditions and discuss barriers and facilitators to collaboration.	A primary barrier was lack of knowledge about the services and policies of each other's systems. Another barrier was differences in philosophy about employment and the special needs of transition-age youth with mental health needs.
[45]	Nicaise et al. (2021) Belgium	Quantitative	19 networks that included 994 services across Belgium.	To evaluate the quality of collaboration between professionals from different services providing care to severe mental illness patients in the Belgian networks, and how inter-individual and interorganizational components of collaboration quality were associated.	Interpersonal collaboration was significantly higher than interorganizational collaboration. Despite the internal consistency of the model, analysis showed that respondents perceived a conflict between client-centered care and leadership in the network. The results reveal two approaches to collaborative service networks, one relying on interpersonal interactions and driven by client needs and another based on formalization and driven by governance procedures. The results reflect a lack of strategy by network leaders for supporting client-centered care and, hence, the persistence of the high level of fragmentation that networks were expected to address. Policy-makers should pay more attention to network formalization and governance mechanisms with a view to achieving effective client-centered outcomes.
[46]	Biringer et al. (2020) Norway	Qualitative	Six service users with complex and severe mental health problems using the outreach team at a community mental health centers (CMHC) and 36 professionals from relevant services.	To explore professionals' and service users' experiences and perceptions of interprofessional collaboration and coordination for service users with complex and severe mental health issues.	Participants described challenges and suggested improvements concerning Distribution of roles, responsibilities, and tasks; Communication; and Knowledge and attitudes. Practice implications: Mental health nurses and other professional helpers should have a particular focus on common aims, clear division of roles, planning and timing of interventions, and communication with other professionals and service users.

[47]	Bogucki et al. (2020) USA	Narrative review	N/A	This review discusses the role of the patient-centered medical home (PCMH) in treating depression, focusing on findings from primary care-based studies and their implications for PCMHs.	The core components of the PCMH are critical elements of depression treatment. Comprehensive care within PCMHs addresses medical and behavioral health concerns, including depression. Psychiatric and psychological care must be flexibly delivered so services remain accessible yet patient-centered. To ensure the quality and safety of treatment, depression symptoms must be consistently monitored. Coordination within and occasionally outside the PCMH is needed to ensure patients receive the appropriate level of care. More research is needed to empirically evaluate depression treatment within PCMHs.
[48]	Connolly et al. (2020) USA	Qualitative	Three external facilitators (EFs) from mental health teams of Veterans Affairs medical centers.	To evaluate Internal Facilitators' use of i-PARIHS facilitation skills, from External Facilitators' perspectives; identify attributes of Internal Facilitators not encompassed within the i-PARIHS skills; and investigate the relative contributions of Internal and External Facilitators during facilitation.	EFs emphasized the importance of IFs having strong project management, team/process, and influencing/negotiating skills. Prior experience in these areas and a mental health background were also beneficial. Personal characteristics (e.g., flexible, assertive) were described as critical, particularly when faced with conflict. EFs discussed the importance of clear delineation of EF/IF roles, and the need to shift facilitation responsibilities to IFs.
[49]	Kebe et al (2020) Canada	Quantitative	Mental Health professionals from four Quebec local health service networks (LHSNs)	This study has two aims: first, to identify variables associated with interprofessional collaboration (IPC) among Quebec mental health (MH) professionals working in MH primary care teams (MH-PCTs) or in specialized service teams (MH-SST); and second, to compare IPC associated variables in MH-PCTs vs MH-SSTs.	Results showed that knowledge integration, team climate and multifocal identification were independently and positively associated with IPC in both MH-PCTs and MH-SSTs. By contrast, knowledge sharing was positively associated with IPC in MH-PCTs only, and organizational support positively associated with IPC in MH-SSTs. Finally, one variable (age) was significantly and negatively associated with IPC in SSTs.

[50]	Menear et al. (2020) Canada	Systematic review	148 unique collaborative mental health care programs included (148 primary trials articles and 430 'sibling' articles)	To identify strategies for engaging patients and families in collaborative mental health care (CMHC) programs for depression and anxiety disorders.	The most common strategies were patient education (87% of programs) and self-management supports (47% of programs). Personalized care planning, shared decision making, and family or peer support were identified in fewer than one third of programs.
[51]	Saraiva et al. (2020) Brazil	Narrative review	48 articles (24 on matrix support and 24 on collaborative care).	This article compares the matrix support model and the collaborative care model.	The main similarity between the models lies in the direct collaborative activities around clinical problems present in the educational support dimension of matrix support and in the dimensions of multidisciplinary care and systematic communication of collaborative care. There are also similarities between the co-management dimension of matrix support and activities from the systematic communication and organizational support dimensions of shared care, such as regular meetings, defining professional roles, and identifying training needs. Structured care and support at the organizational level should be encouraged under certain conditions, but the emphasis on the political aspect of the clinic and the democratization of relationships between professionals are examples of advancement of matrix support in relation to collaborative care.
[52]	Skyberg & Innvaer (2020) Norway	Qualitative	Three interprofessional teams in the field of mental health and substance use in Norway. 8–14 employees per team with professional backgrounds in nursing, occupational therapy, psychiatry, clinical psychology, social	This paper examines interprofessional teamwork and how different health and social professions work together as a group.	The dynamics of interprofessionalism point to three logics of professional boundaries and roles: drawing boundaries, boundary blurring and the complementary of professional roles. Each logic has its purpose and function. All three logics operate in the same context, sometimes within the same situation. Combining the three logics offer a comprehensive view of interprofessionalism.

			education and social work.		
[53]	Coffey et al. (2019) United Kingdom	Mixed methods	19 mental health wards in six service provider sites in England and Wales. This included a survey using established standardised measures of service users (n = 301) and staff (n = 290) and embedded case studies involving interviews with staff, service users and carers (n = 76).	To ascertain the views of service users, carers and staff in acute inpatient wards on factors that facilitated or acted as barriers to collaborative, recovery-focused care.	For service users, when recovery-oriented focus was high, the quality of care was rated highly, as was the quality of therapeutic relationships. For staff, there was a moderate correlation between recovery orientation and quality of therapeutic relationships, with considerable variability. Staff members rated the quality of therapeutic relationships higher than service users did. Staff accounts of routine collaboration contrasted with a more mixed picture in service user accounts. Definitions and understandings of recovery varied, as did views of hospital care in promoting recovery. Managing risk was a central issue for staff, and service users were aware of measures taken to keep them safe, although their involvement in discussions was less apparent.
[54]	Johnson & Mahan (2019) USA	Qualitative	32 behavioral health provider's.	This qualitative investigation sought to add to the limited research on behavioral health providers and interprofessionalism by answering the following research question: What are the attitudes of behavioral health providers who are engaged in interprofessional clinical	Two major themes: (1) benefits (to the profession and client) and (2) collaboration (collaboration experience and consultation). A strengths, weaknesses, opportunities, and threats analysis was used to operationalize the findings and develop implications.

				collaboration?	
[55]	Sather et al. (2019) Norway	Qualitative	Ten former patients from five communities who had previously experienced transition into and out of inpatient services.	This study explores former patients' views of pathways in transition between district psychiatric hospital centres (DPCs) and community mental health services.	The informants shared their experiences on issues promoting and preventing successful care pathways in mental health. Four main paired themes were identified: (a) patient participation/activation/empowerment versus paternalism and institutionalization, (b) patient-centred care versus care interpreted as humiliation, (c) interprofessional collaboration or teamwork versus unsafe patient pathways in mental health services, and (d) sustainable integrated care versus fragmented, noncollaborative care.
[56]	Bradley & Green (2018) United Kingdom	Qualitative	Professionals and family members from a large mental health and learning disability National Health Service (NHS) organization.	This study aimed to better understand how the family caregivers of those diagnosed with severe mental illness are currently involved in decision making, particularly decisions about treatment options including prescribed medication.	Themes included the definition of involvement and “rules of engagement.” Staff members are gatekeepers for family involvement, and the process is not democratic. Family and staff ascribe practical, rather than recovery-oriented roles to family, with pre-occupation around notions of adherence.
[57]	Jafelice & Marcolan (2018) Brazil	Qualitative	27 professionals from nine CAPS (Psychossocial Care Centers).	To analyze the conceptions of the CAPS workers on the different forms of disciplinary integration and as they happened in the reality of the services of the city of São Paulo/SP.	There were difficulties in conceptualizing modalities of disciplinary integration (multiprofessional, interprofessional and transprofessional work) and little problematization in the reality of workers. Few questions regarding specific practices of each area were made.
[58]	Tomizawa et al. (2017) Japan	Quantitative	136 assessment tools were retrieved for review of the Collaborative Practice Assessment Tool (CPAT).	To revise and validate an interprofessional scale to assess the quality of teamwork in inpatient psychiatric units and to use it multi-nationally.	Exploratory factor analysis yielded five factors with 21 items: 'patient/community centred care', 'collaborative communication', 'interprofessional conflict', 'role clarification', and 'environment'. High overall internal consistency, reproducibility, adequate face validity, and reasonable construct validity were shown in the USA and Japan.

[59]	Avny et al. (2016) Israel	Narrative review	N/A	To present a literature review of collaborative enterprises between psychiatrists and primary care physicians in Israel and other countries. Also described are local psychiatric liaison initiatives in Israel, as well as landmark studies of collaborative psychiatric care.	These studies demonstrate the superiority of community psychiatric liaison models in the treatment of patients suffering from depressive anxiety disorders and somatization disorder. In light of the mental health reform currently underway in Israel, it is important to develop, implement and assess such liaison models.
[60]	Millar et al. (2016) United Kingdom	Concept analysis	134 retained citations.	This study sought to explore and analyse the concept of service user involvement as used within the field of mental health care.	Five key attributes of service user involvement within the context of mental health care were identified: a person-centred approach, informed decision making, advocacy, obtaining service user views and feedback and working in partnership.
[61]	Sapag et al. (2016) Canada	Mixed methods	Local stakeholders from three public health networks in Mexico, Nicaragua and Chile (decision-makers in key informant interviews, front-line clinicians in focus groups and other stakeholders through a survey).	This study examined Latin American evaluation needs regarding the development of a collaborative mental health care (CMHC) evaluation framework as seen by local key health-care leaders and professionals. Potential implementation challenges and opportunities were also identified.	Participants recognized a strong need to evaluate different areas of CMHC in Latin America, including access, types and quality of services, human resources and outcomes related to mental disorders, including addiction. A priority was to evaluate collaboration within the health system, including the referral system. Issues of feasibility, including the weaknesses of information systems, were also identified.
[62]	Nadeem et al. (2014) USA	Systematic review	20 articles selected for inclusion.	The purpose of this review is to characterize the state of the evidence on Learning Collaboratives (LC) in mental health care.	The LCs in this review reported including, on average, seven components, most commonly in-person learning sessions, Plan-Do-Study-Act (PDSA) cycles, multidisciplinary QI teams, and data collection for QI.

[63]	Dadich et al. (2013) Australia	Qualitative	20 consumers from a community care programme for people in New South Wales (NSW), Australia.	This article analyses an example from the mental health Housing and Accommodation Support Initiative in Australia	The research found they worked effectively in care planning when the planning was consumer-driven; when there was active participation from consumers, non-clinical and clinical case managers; and when planning was treated as a process, with incremental goals, reflective practice, as well as shared understanding and commitment to the collaboration.
[64]	DeJesse (2013) USA	Qualitative	22 professionals who specialize in the treatment of eating disorders (12 mental health providers, 10 registered dieticians).	The goal of this study was to build on existing knowledge about collaboration between mental health providers (MHP) and nutritionists (NT)	Seven themes emerged and were described as Best Practices in Mental Health Professional–Nutritionist Collaborations: Cultivate a provider network; Accept differences; Build good fences; Communicate; Value counterparts; Seek team consensus; Educate
[65]	Goodrich et al. (2013) USA	Narrative review	A total of 74 articles were identified for inclusion in this literature review.	This paper presents a critical review of the recent literature published about the topic of Collaborative care models (CCMs) for mental health in primary care settings, with particular emphasis on highlighting literature relevant to the implementation of this treatment model in routine practice.	Collaborative care models (CCMs) provide a pragmatic strategy to deliver integrated mental health and medical care for persons with mental health conditions served in primary care settings. CCMs are team-based interventions to enact system-level redesign by improving patient care through organizational leadership support, provider decision support, and clinical information systems as well as engaging patients in their care through self-management support and linkages to community resources. The model is also a cost-efficient strategy for primary care practices to improve outcomes for a range of mental health conditions across populations and settings. CCMs can help achieve integrated care aims under healthcare reform yet organizational and financial issues may affect adoption into routine primary care. Notably, successful implementation of CCMs in routine care will require alignment of financial incentives to support systems redesign investments, reimbursements for mental health providers, and adaptation across different practice settings and infrastructure to offer all CCM components.
[66]	Frounfelker et al. (2012) USA	Narrative review	The Thresholds psychiatric rehabilitation	This paper identifies potential barriers to academic-provider research collaborations and	Realistic resource allocation and training, a thorough understanding of the service model and consumer characteristics, systemic and bidirectional communication and concrete plans for postproject continuation are necessary

			agency and the Dartmouth Psychiatric Research Center partnership.	provides guidelines to overcome these obstacles.	at all project phases. Conclusions: A shared decision-making framework is essential for effective academic institution and community mental health agency collaborations and can facilitate long-term sustainability of novel interventions.
[67]	Gehart (2012) USA	Narrative review	N/A	This article outlines a collaborative, appreciative approach for working in recovery-oriented contexts.	The model outlined in this article includes an overview of the recovery partnership (i.e., therapeutic relationship), mapping recovery (i.e., assessment and case conceptualization), recovery planning (i.e., treatment planning), facilitating recovery (i.e., intervention), accessing resources (i.e., case management), recovery maintenance, and service contexts as well as a case study.

N/A: Not applicable
